# Accelerated biological aging in COVID-19 patients

**DOI:** 10.1038/s41467-022-29801-8

**Published:** 2022-04-19

**Authors:** Xue Cao, Wenjuan Li, Ting Wang, Dongzhi Ran, Veronica Davalos, Laura Planas-Serra, Aurora Pujol, Manel Esteller, Xiaolin Wang, Huichuan Yu

**Affiliations:** 1 Department of Oncology, Guizhou Provincial People’s Hospital, Guiyang, Guizhou China; 2 Guangdong Institute of Gastroenterology, Guangdong Provincial Key Laboratory of Colorectal and Pelvic Floor Disease, The Sixth Affiliated Hospital, Sun Yat-sen University, Guangzhou, Guangdong China; 3 Department of Colorectal Surgery, The Sixth Affiliated Hospital, Sun Yat-sen University, Guangzhou, Guangdong China; 4 Department of Pulmonary and Critical Care Medicine, The Third Affiliated Hospital, Sun Yat-sen University, Guangzhou, Guangdong China; 5 Research & Development, Thermo Fisher Scientific Inc., Los Angeles, CA USA; 6 Department of Pharmacology, College of Medicine, University of Arizona, Tucson, AZ USA; 7 Key Laboratory of Biochemistry and Molecular Pharmacology, Department of Pharmacology, Chongqing Medical University, Chongqing, China; 8 Josep Carreras Leukaemia Research Institute (IJC), Barcelona, Catalonia Spain; 9 Neurometabolic Diseases Laboratory, Bellvitge Biomedical Research Institute (IDIBELL), Barcelona, Catalonia Spain; 10 Center for Biomedical Research on Rare Diseases (CIBERER), ISCIII, Madrid, Spain; 11 Institucio Catalana de Recerca i Estudis Avancats (ICREA), Barcelona, Catalonia Spain; 12 Centro de Investigación Biomédica en Red de Cancer (CIBERONC), Madrid, Spain; 13 Physiological Sciences Department, School of Medicine and Health Sciences, University of Barcelona (UB), Barcelona, Catalonia Spain

**Keywords:** DNA methylation, Viral infection, Ageing

## Abstract

Chronological age is a risk factor for SARS-CoV-2 infection and severe COVID-19. Previous findings indicate that epigenetic age could be altered in viral infection. However, the epigenetic aging in COVID-19 has not been well studied. In this study, DNA methylation of the blood samples from 232 healthy individuals and 413 COVID-19 patients is profiled using EPIC methylation array. Epigenetic ages of each individual are determined by applying epigenetic clocks and telomere length estimator to the methylation profile of the individual. Epigenetic age acceleration is calculated and compared between groups. We observe strong correlations between the epigenetic clocks and individual’s chronological age (*r* > 0.8, *p* < 0.0001). We also find the increasing acceleration of epigenetic aging and telomere attrition in the sequential blood samples from healthy individuals and infected patients developing non-severe and severe COVID-19. In addition, the longitudinal DNA methylation profiling analysis find that the accumulation of epigenetic aging from COVID-19 syndrome could be partly reversed at late clinic phases in some patients. In conclusion, accelerated epigenetic aging is associated with the risk of SARS-CoV-2 infection and developing severe COVID-19. In addition, the accumulation of epigenetic aging from COVID-19 may contribute to the post-COVID-19 syndrome among survivors.

## Abstract (web summary)

Age is a risk factor for SARS-CoV-2 infection and severe disease. Here the authors perform DNA methylation analyses in whole blood from COVID-19 patients using established epigenetic clocks and telomere length estimators, and describing correlations between epigenetic aging and the risk of SARS-CoV-2 infection and severe disease.

## Introduction

The ongoing pandemic of coronavirus disease 2019 (COVID-19) due to severe acute respiratory syndrome-corona virus 2 (SARS-CoV-2) infection is a rapidly developing global emergency which already claimed over 170 million cases and 3.8 million lives worldwide as of June 13rd, 2021 (WHO Coronavirus Situation Report, 2021)^1^. The incidence of severity and death in COVID-19 patients has been reported to increase with age^2–5^. Chronological age has been proved to be one of the major risk factors for developing severe COVID-19 and death^6^, and this risk is independent of other age-related comorbidities like diabetes, cardiovascular diseases, or obesity^7^. Therefore, understanding the biological aging of COVID-19 in severe cases versus non-severe patients could be quite useful to model and predict the disease progression with other laboratory assays^8^.

Aging is a biological process related to diseases and mortality. The biological process in aging is reflected by molecular hallmarks, which include epigenetic modifications and telomere attrition^9–11^. DNA methylation correlates with aging process and can be used to estimate epigenetic aging across tissues^12–14^. The deviation between DNA methylation age (DNAm age) and chronological age has been proposed as a biomarker for aging and has been related to risk and survival outcomes in age-related diseases^15,16^. Previous work showed that the epigenetic landscape of host cell is altered during HIV^17–19^ and coronavirus^20–23^ infection, including SARS-CoV-2. In addition, telomere attrition in leukocytes is another hallmark of aging and associated with increased risk of aging-related diseases and human life span^24–26^, and a DNAm-based telomere length estimator (DNAm TL) has been developed^27^. Interestingly, telomere length and the epigenetic clock do not correlate with one another, suggesting that DNAm age and TL measure different aspects of biological aging^28,29^. Recently, epigenetic age at the moment of the infection has been reported to be associated with COVID-19^30–32^. However, little is known about the epigenetic aging during SARS-CoV-2 infection and in COVID-19 survivors and whether this factor might help predict the risk of developing severe COVID-19.

In this study, we estimated the epigenetic age of the whole blood in COVID-19 patients and healthy individuals using the previously established epigenetic clocks (Hannum, Horvath, PhenoAge, skinHorvath and GrimAge clocks)^12,33–36^ and telomere length estimator^27^. We defined epigenetic age acceleration for each case by comparing the individual’s epigenetic age with chronological age to assess whether the accelerated or dysfunctional epigenetic aging is associated with SARS-CoV-2 infection and the severity of COVID-19 syndrome.

## Results

### Assessment of epigenetic age in COVID-19 patients

To study epigenetic aging in the whole blood and its correlation with non-severe and severe COVID-19, we conducted a genome-wide DNA methylation study on whole blood collected from 232 healthy individuals, 194 non-severe and 213 severe COVID-19 patients in the combined set to calculate their DNAm age and TL using five different epigenetic clocks and DNAm TL estimator. The COVID-19 patients were not significantly different from the healthy individuals in terms of age and sex (Supplementary Table 1).

**Fig. 1:**
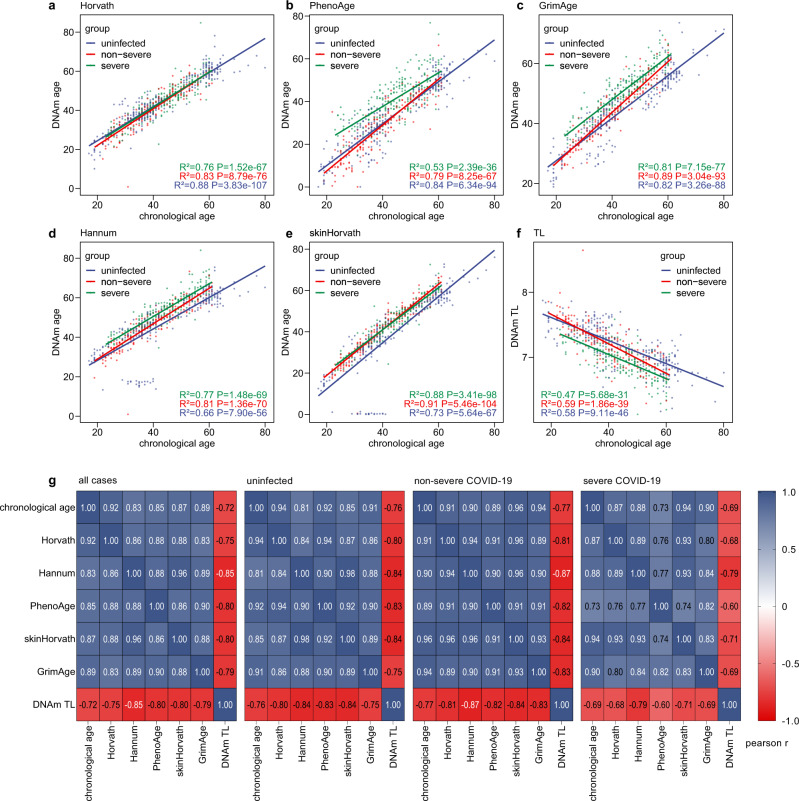
Assessment of epigenetic clocks and DNA methylation-based telomere length estimator in patient cohorts. **a**–**e** Correlation of chronological age with five epigenetic clocks (**a** Horvath; **b** Hannum; **c** PhenoAge; **d** skinHorvath; **e** GrimAge) and DNA methylation-based telomere length (**f** TL) estimator in the peripheral blood from 232 healthy individuals, 194 non-severe and 213 severe COVID-19 patients. **g** Heatmap presents the matrix of Pearson correlation coefficients among chronological age, DNAm ages determined by each epigenetic clock, and DNAm TL in the whole, healthy, non-severe and severe sets. Source data are provided as a Source Data file.

We calculated the Pearson correlation coefficient among individual chronological age, epigenetic clocks and DNAm TL in the combined set of samples. As shown in Fig. 1, all clocks had a strong correlation with the chronological age in the whole set and each sample group (*r* > 0.8, *p* < 0.0001), though the PhenoAge clock showed weaker correlations with chronological age in the severe COVID-19 group (*r* = 0.73, *p* < 0.0001). In addition, all clocks were strongly correlated with one another in all groups (*r* > 0.8, *p* < 0.0001; Fig. 1 and Supplementary Fig. 1), except that PhenoAge clock showed weaker correlations with other clocks in the severe COVID-19 group (*r* = 0.73 ~ 0.77, *p* < 0.0001). DNAm TL was negatively correlated with chronological and DNAm ages in the whole set and all groups (*r* = −0.59 ~ −0.90, *p* < 0.0001; Fig. 1). Interestingly, DNAm TL showed a stronger linear correlation with DNAm ages compared to chronological age (Fig. 1 and Supplementary Fig. 2).

### Accelerated epigenetic aging in SARS-CoV-2 infection

We initially assessed the DNAm ages and TLs of the blood samples and found an older DNAm age in the COVID-19 patients for Horvath, Hannum, skinHorvath and GrimAge clocks compared to the healthy individuals (*p* < 0.05; Supplementary Fig. 3). To adjust for the bias due to individual chronological age, we calculated epigenetic age acceleration for each sample. Individuals with COVID-19 were estimated to have significant DNAm age acceleration for Hannum, PhenoAge, skinHorvath and GrimAge clocks and significant DNAm TL attrition acceleration compared with healthy individuals (all *p* < 0.0001; Supplementary Fig. 4). The same phenomena were observed in the young (age < 50) and old (age ≥ 50) populations (Supplementary Fig. 5). Together, we found an accelerated epigenetic aging in patients with SARS-CoV-2 infection.

### Accelerated epigenetic aging and risk of developing severe COVID-19

**Fig. 2:**
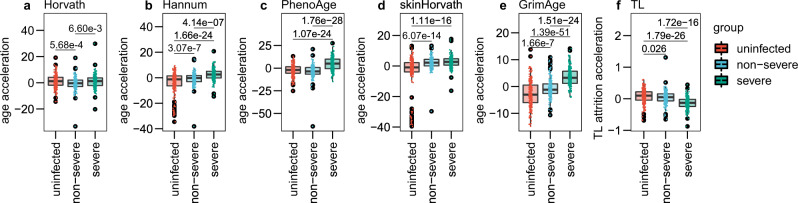
Accelerated epigenetic aging in non-severe and severe COVID-19 patients. Distribution of DNAm age acceleration (**a**–**e**, five epigenetic clocks) and telomere attrition acceleration (**f**, TL) in the peripheral blood from 232 healthy individuals, 194 non-severe and 213 severe COVID-19 patients. The y-axis shows the epigenetic age acceleration after adjusting for cell-type fractions (i.e., residual of regressing the epigenetic age acceleration on cell-type fractions). The *p* value for each t-test is shown above the corresponding line. In the box plots, the lower and upper hinges indicate the 25th and 75th percentiles, and the black line within the box marks the median value. The whiskers extend from the hinges to the largest and smallest values no further than 1.5× inter-quartile range from the hinges, and the points beyond the end of whiskers indicate outliers. Source data are provided as a Source Data file.

Next, we analyzed the association of COVID-19 severity with epigenetic aging. We found individuals with severe COVID-19 had significant DNAm age acceleration for all epigenetic clocks and DNAm TL attrition acceleration compared with healthy individuals (all *p* < 0.0001; Fig. 2). In addition, severe COVID-19 patients had significant DNAm age acceleration for Horvath, Hannum, PhenoAge and GrimAge clocks and significant DNAm TL attrition acceleration compared with non-severe COVID-19 patients (all *p* < 0.05; Fig. 2). Moreover, non-severe COVID-19 patients had significant DNAm age acceleration for Horvath, Hannum, skinHorvath and GrimAge clocks and significant DNAm TL attrition acceleration compared with healthy individuals (all *p* < 0.05; Fig. 2). Among the five epigenetic clocks, the statistical difference in the pairwise test for GrimAge clock was most significant. In addition, we found that COVID-19 patients developing pneumonia had significantly accelerated epigenetic aging compared with those not developing pneumonia (Supplementary Fig. 6). Together, we found an increasing acceleration of epigenetic aging in the sequential samples of healthy, non-severe and severe groups.

### Dynamic acceleration of epigenetic aging across COVID-19 disease phases

To explore the role of COVID-19 in epigenetic age acceleration, we analyzed the dynamic acceleration of epigenetic age across COVID-19 disease phases in the Bernarde et al’s longitudinal cohort^37^ with six COVID-19 cases and six uninfected controls. Clinical disease phases were defined based on inflammatory markers and ventilation needs, which reflected temporal disease severity and distinguish between incremental and recovering disease stages, according to the WHO ordinal scale described by Bernardes et al.^37^.

**Fig. 3:**
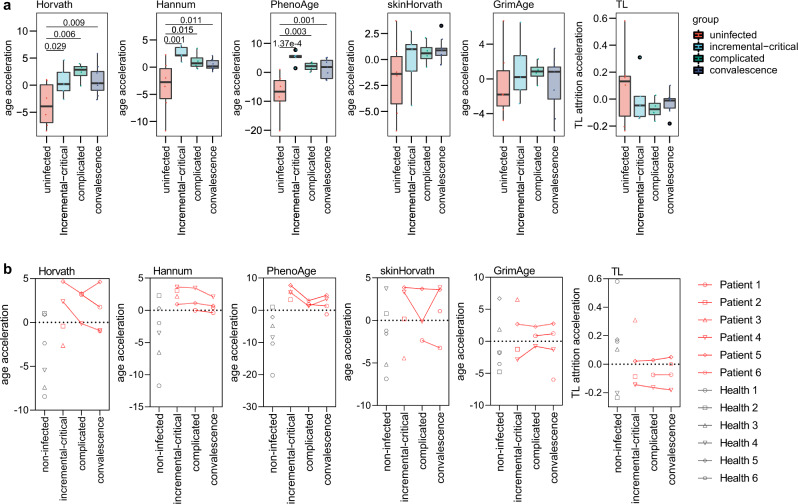
Epigenetic aging across COVID-19 disease phases. **a** Acceleration of DNAm aging (five epigenetic clocks) and telomere attrition of the blood samples collected from uninfected control and patients at each clinical disease phase. **b** Dynamic change of epigenetic age acceleration in each individual across COVID-19 disease phases. The *p* value for each t-test is shown above the corresponding line. In the box plots, the lower and upper hinges indicate the 25th and 75th percentiles, and the black line within the box marks the median value. The whiskers extend from the hinges to the largest and smallest values no further than 1.5 × inter-quartile range from the hinges, and the points beyond the end of whiskers indicate outliers. Source data are provided as a Source Data file.

We assessed the DNAm ages and TLs of the samples collected from each disease phase and found an increasing acceleration of epigenetic aging at the initial phases of COVID-19, and this age acceleration could be partly reversed at later phases (Fig. 3a). Specifically, we found an increasing acceleration of Horvath age at the initial two disease phases and this acceleration was partly reversed in the upcoming convalescence phase. Similarly, Hannum and PhenoAge clocks were accelerated at the initial stage of incremental and critical disease phase and reversed in the upcoming complicated and convalescence phases. In addition, an increasing attrition acceleration of DNAm age at the initial two disease phases was found to be partly reversed in the upcoming convalescence phase, although the differences between every two phases were not statistically significant.

Furthermore, we tracked the dynamic changes of DNAm ages and TLs during the clinical disease phases in individual patients and observed similar result to the group-wise comparison. The accelerated DNAm ages at initial phases were partly reversed at the later phases in some COVID-19 patients by using the five epigenetic clocks, while the reversible outcome of accelerated attrition of DNAm TL from initial to later phases was found only in one patient (Fig. 3b). Therefore, we speculated that COVID-19 syndrome might accelerate epigenetic aging in SARS-CoV-2 infected patients, and this process would be partly reversible in the clinical disease phases.

## Discussion

In this study, we took advantage of prior indication that epigenetic age could be altered in the presence of viral infections^17,20,38^ and from the fact that shorter telomeres have been associated with the risk of developing COVID-19 with worse outcomes^32^. We used five epigenetic clocks and one telomere length estimator to determine the epigenetic age of the whole blood in COVID-19 patients and healthy individuals. We observed strong correlation of the epigenetic clocks with the chronological age and increasing acceleration of epigenetic aging in the blood samples of healthy individuals and COVID-19 patients. Our epigenetic study with consistent findings from different epigenetic clocks provides evidence for the association of accelerated epigenetic aging with the risk of SARS-CoV-2 infection and developing severe COVID-19. In addition, we found a reversible influence of COVID-19 syndrome on epigenetic aging in some COVID-19 patients with the longitudinal DNA methylation profiling analysis. Overall, these findings suggest that COVID-19 may perturb the epigenetic clock and telomere length. Although the epigenetic clocks and telomere length are known as independent^28,29^, DNAm aging was parallelled by a telomere shortening in all the observations.

DNA methylation-based estimator of telomere length was shown to have a relatively weak correlation with PCR-based telomere length that is recognized as an accurate quantification^36^. Therefore, PCR assays are needed to validate the correlation between telomere length and COVID-19. Mongelli et al. applied PCR assay to quantify telomere length and found a telomere shortening in the post-COVID-19 survivors^39^. Although some of our findings could be supported by Mongelli, the association of telomere attrition with severe COVID-19 and dynamic change of telomere length across disease phases need to be confirmed in a longitudinal cohort with PCR assays.

Epigenetic changes have been reported to be associated with the SARS-CoV-2 infection and unfavorable COVID-19 outcomes, including the methylation regulation of Angiotensin Converting Enzyme 2 (*ACE2*)^40^, interferon-related pathways^23^, and immune response genes^23,41^. Considering the DNA methylation changes in SARS-CoV-2 infection that may affect the expression of metabolic process and epigenetic aging of COVID-19^41^, a further question could be whether the accelerated epigenetic aging exists before the first viral contact, worsening progressively up or coming back to normal during the convalescence phases and post-COVID-19 period. Our analysis of epigenetic clocks and telomere shortening indicates an increasing age acceleration at the initial phases of COVID-19 and this could be partly reversed at later phases. We speculated that COVID-19 syndrome might accelerate epigenetic aging in SARS-CoV-2-infected patients based on these findings. Our longitudinal DNA methylation profiling analysis indicates that the influence of COVID-19 syndrome on epigenetic aging in peripheral blood could be reversed in some patients. However, additional experiments are necessary to validate these findings and elucidate the underlying mechanism.

In the current study, we found accelerated epigenetic aging could serve as a predicting biomarker for severe COVID-19 that requires hospitalization with increased mortality. However, a recent study found that the association of accelerated epigenetic aging with severe COVID-19 varied among different epigenetic clocks and datasets^42^, which might be attributed to the small sample size in some datasets, unspecified severity of COVID-19 cases, and the different estimation method of age acceleration. Interestingly, the difference between severe COVID-19 patients and other individuals in the GrimAge clock was most significant among the five epigenetic clocks in our cohort. The strong stratification in the severity of COVID-19 may come from the correlation between GrimAge clock and smoking-associated changes that were shown to be a prognostic factor for severe illness and mortality in COVID-19 patients^43–45^.

Of note, there is a lack of molecular biomarkers potentially valuable in monitoring post-COVID-19 syndrome that will require long-term assistance among the millions of COVID-19 survivors^46^. Based on our findings, we speculate that the accumulation of epigenetic aging and telomere attrition after SARS-CoV-2 infection might contribute to the post-COVID-19 syndrome, and irreversible epigenetic aging might be served as a biomarker for the risk of developing post-COVID-19 syndrome.

It is noteworthy that this study has some limitations. First, our longitudinal analysis indicates the recovery of accelerated epigenetic aging occurred in some patients, though none of the post-COVID-19 survivors with post-COVID-19 syndrome was included in our study. In addition, the limited cases were included in the longitudinal analysis, and the findings require validation in a larger and more diverse longitudinal cohort. Next, the causal relationship between COVID-19 and accelerated epigenetic aging remains unanswered in the current study. A longitudinal cohort with sustaining follow-up from the time point before SARS-CoV-2 infection to post-COVID-19 phase would be helpful to address this question, though it would be challenging to conduct a study with this design. A long-term follow-up of crowdsourced populations^47,48^ may facilitate the longitudinal research and provide valuable evidence. Furthermore, we cannot rule out the potential confounding effects of chronic inflammation, oxidative stress in respiratory failure and prior medication use. Finally, the application of epigenetic aging markers in predicting COVID-19 outcomes is limited by the cost and accessibility of methylation arrays. However, representative age-related CpG methylations could be applied by using qPCR-based assays and pyrosequencing^39,49,50^. We anticipate that epigenetic aging-related markers would be useful to predict disease progression in COVID-19 with other laboratory assays^8,51^.

In conclusion, our results indicate that accelerated epigenetic aging is associated with the risk of SARS-CoV-2 infection and developing severe COVID-19. In addition, COVID-19 could exert influence on the epigenetic clock and telomere attrition and accelerate the epigenetic aging, which may contribute to the post-COVID-19 syndrome. However, this influence is reversible in some patients. Together with other laboratory assays and clinical characteristics, it would be helpful to identify the patients with high risk of developing severe COVID-19 and post-COVID-19 syndrome.

## Methods

### Study design and patients

This study was designed to compare the DNAm age and TL in COVID-19 patients and healthy individuals and determine the dynamic change of DNAm age and TL across COVID-19 disease phases. This study included whole blood samples of 407 COVID-19 patients collected from fourteen hospitals in Spain^23^, six COVID-19 patients from Bernardes et al’s longitudinal cohort in Germany^37^ and 232 healthy individuals from previous studies with well-characterized populations^52–56^. The included COVID-19 patients did not present the risk factors of comorbidities as we previously described^23^. The whole blood samples of healthy individuals were collected before 2019 to ensure they had never been exposed to SARS-CoV-2. The healthy individuals and COVID-19 patients did not present significant differences in age and gender (Supplementary Table 1). The protocol of this study was approved by the institutional ethics review board of Josep Carreras Leukaemia Research Institute. Written informed consent was obtained from all participants. We conducted this study in compliance with the principles of the Declaration of Helsinki.

### Methylation array data processing

DNA from the whole blood samples in all cases were hybridized to the Illumina Infinium MethylationEPIC Beadchip (EPIC array) that interrogates almost 850,000 CpG sites of the human genome. The “minfi” R package^57^ and Combat tool were used to perform raw data processing, batch effect correction, and data analysis as we previously described^15,58–60^. The methylation β value for each CpG site in each sample was calculated to represent the methylation level.

### DNA methylation age calculation

We determined the DNAm age with the blood-based epigenetic clocks, including the Horvath clock with 353 CpGs based on multiple tissue types^12^, the Hannum clock with 71 CpGs identified in blood DNA samples^33^, the PhenoAge clock based on 513 CpGs derived from whole blood^34^, the skinHorvath clock that integrated the dataset of whole blood with skin, endothelial cells, mouth mucosa and saliva^35^, and the GrimAge clock using 1030 CpGs associated with smoking packyears and physiological risk factors^36,61^.

Although the EPIC array lacks some of the CpGs in the Hannum and Horvath clocks due to the difference of EPIC array with HM450 array that these clocks rely on, missing clock CpGs on the EPIC array was demonstrated to not substantially affect the accuracy of the DNAm age estimation using blood sample by McEwen et al.^62^ or epithelial tissue in our previous work^15^. The deviation between epigenetic age and chronological age, also known as epigenetic age acceleration, was calculated for every sample based on the residuals from regressing the DNAm age or TL on chronological age, as described by McEwen et al.^62^.

### Telomere length estimation

To explore the dynamic change of telomere length in the COVID-19 disease process, we used a DNA methylation-based estimator for telomere length based on the Horvath’s Elastic Net regression model^27^. The deviation between DNAm TL and chronological age, defined as DNAm TL attrition acceleration, was calculated for every sample based on the residuals from regressing the DNAm TL on chronological age.

### Cell composition estimation

Based on the accumulating evidence reporting the change of immune cell composition in severe COVID-19 patients,^37,63,64^ we estimated the cell counts with the ‘meffil’ tool^65^ and performed cell composition adjustment to control the impact on epigenetic age acceleration estimation.

### Statistical methods

The correlation among DNAm age, DNAm TL and chronological age of the samples was calculated with the Pearson correlation coefficient. A linear regression model was applied to determine the relationship between the epigenetic age and chronological age at the time of sample collection. All statistical analysis was performed using R Statistical Software (version 3.6.1). *P* values < 0.05 were considered statistically significant.

### Reporting summary

Further information on research design is available in the Nature Research Reporting Summary linked to this article.

## Supplementary information

Supplementary Information

Reporting Summary

## Data Availability

The complete DNA methylation raw data of the severe and non-severe COVID-19 cases have been deposited on the GEO repository under accession number GSE168739, and the clinical outcomes of these cases are not publicly available for data privacy but are available from Dr. Manel Esteller (mesteller@carrerasresearch.org) upon request for research collaboration. The timeframe for response to data access requests is 30 days. There are no restrictions on the reuse of data. In addition, the DNA methylation raw data of the longitudinal cohort and healthy individuals analyzed in this study were available at GEO with identifiers of GSE161678, GSE149318, GSE123914, GSE145254, GSE118144 and GSE141682. Source data are provided with this paper.

## Source data

Source Data
