# Supplementary material for: Accelerated biological aging in COVID-19 patients
Source: Nat Commun. 2022 Apr 19;13:2135. doi: 10.1038/s41467-022-29801-8 (PMC9018863; doi:10.1038/s41467-022-29801-8)
Supplement: Supplementary file 1 — Supplementary Information [file 41467_2022_29801_MOESM1_ESM.pdf]

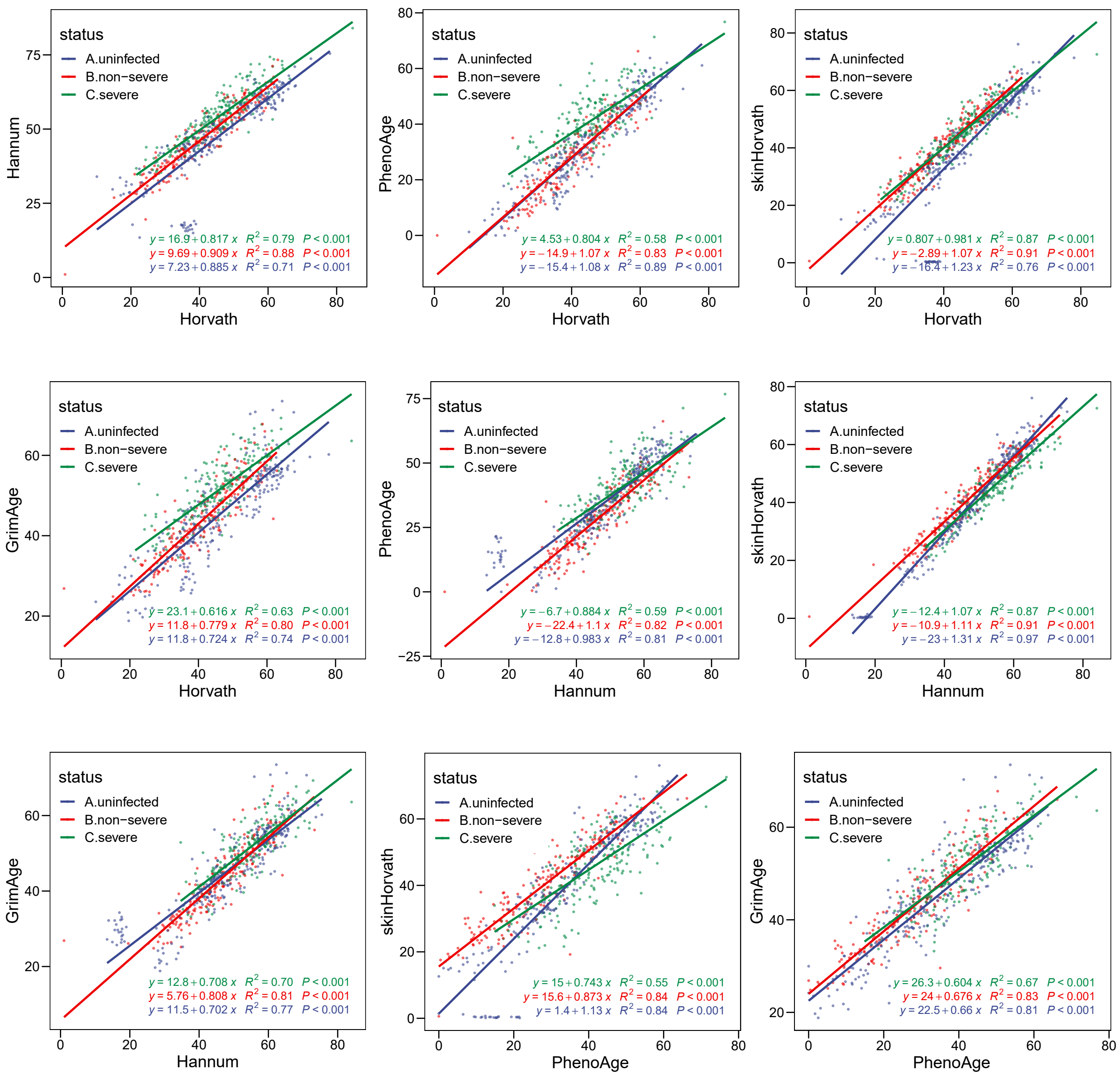

Supplementary figure 1 Correlation between epigenetic clocks

The scatter dot plots with regression lines show the associations between every two epigenetic clocks of Horvath, Hannum, PhenoAge, skinHorvath and GrimAge clocks in uninfected control and non-severe and severe COVID-19 patients.

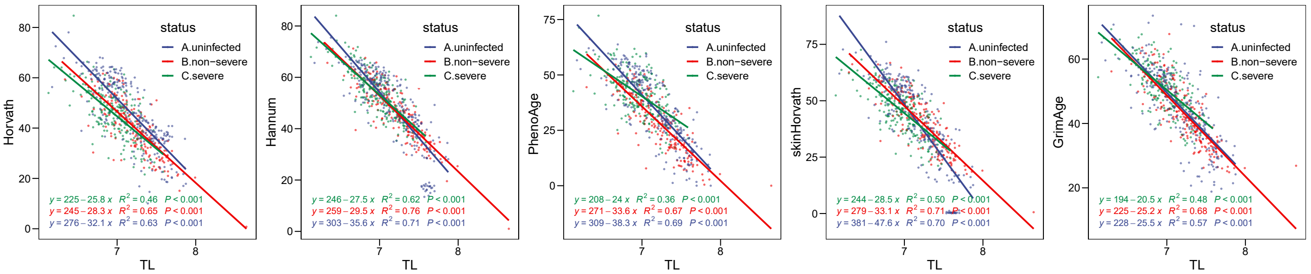

Supplementary figure 2 Association of telomere length with chronological and epigenetic ages in healthy controls, severe and non-severe COVID-19 patients

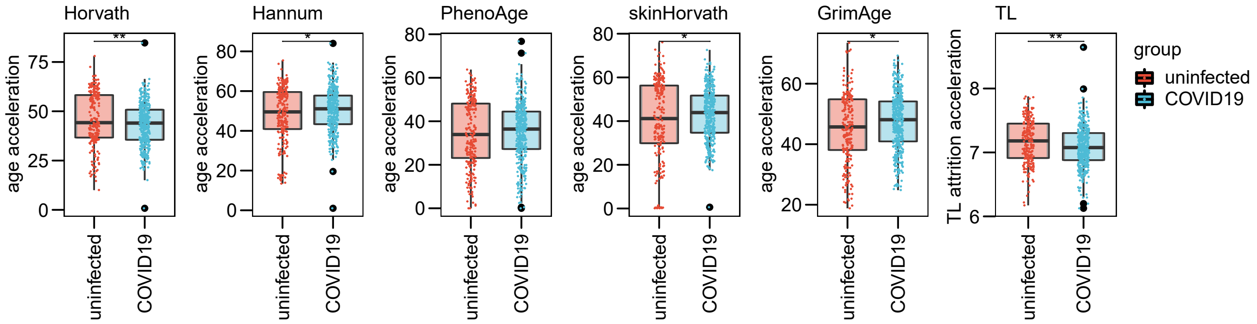

Supplementary figure 3 DNAm age and telomere length in healthy individuals and COVID-19 patients

The statistical significance for the t-test (*, p < 0.05; **, p <0.01; ***, p < 0.001; ****, p < 0.0001) is shown above the corresponding line.

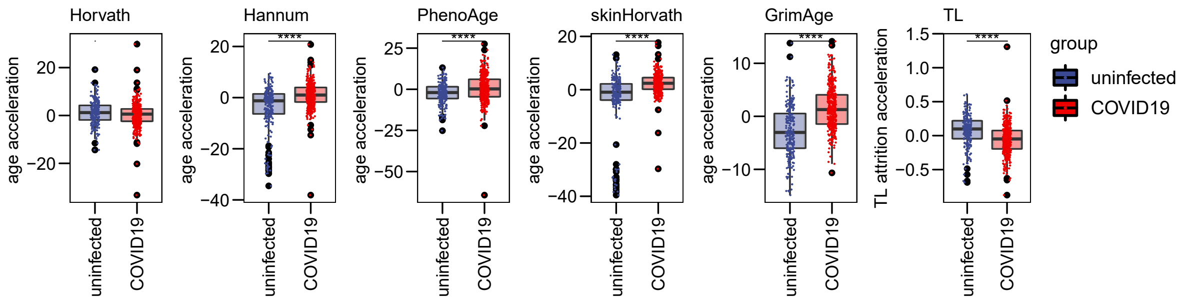

Supplementary figure 4 Epigenetic age acceleration in healthy individuals and COVID-19 patients

The statistical significance for the t-test (*, $p < 0.05$; **, $p < 0.01$; ***, $p < 0.001$; ****, $p < 0.0001$) is shown above the corresponding line.

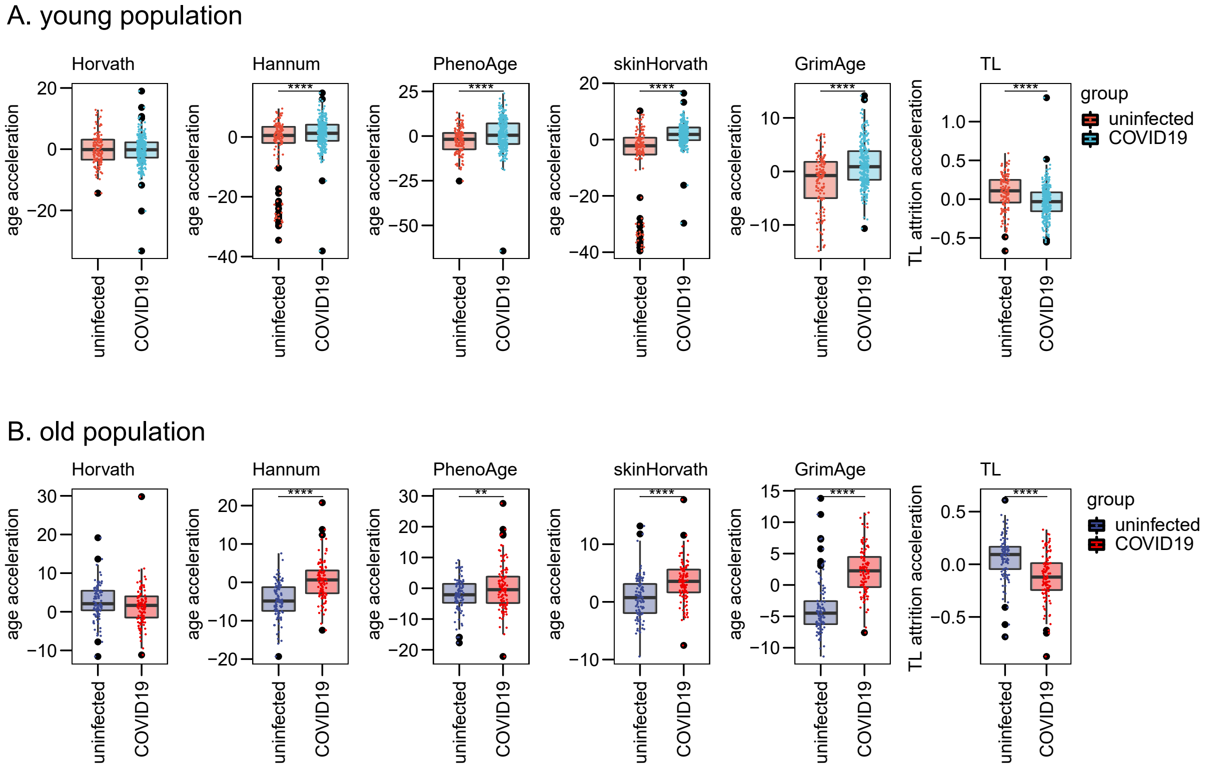

Supplementary figure 5 Epigenetic age acceleration in young (A) and old (B) populations

The statistical significance for the t-test (*, p < 0.05; **, p <0.01; ***, p < 0.001; ****, p < 0.0001) is shown above the corresponding line.

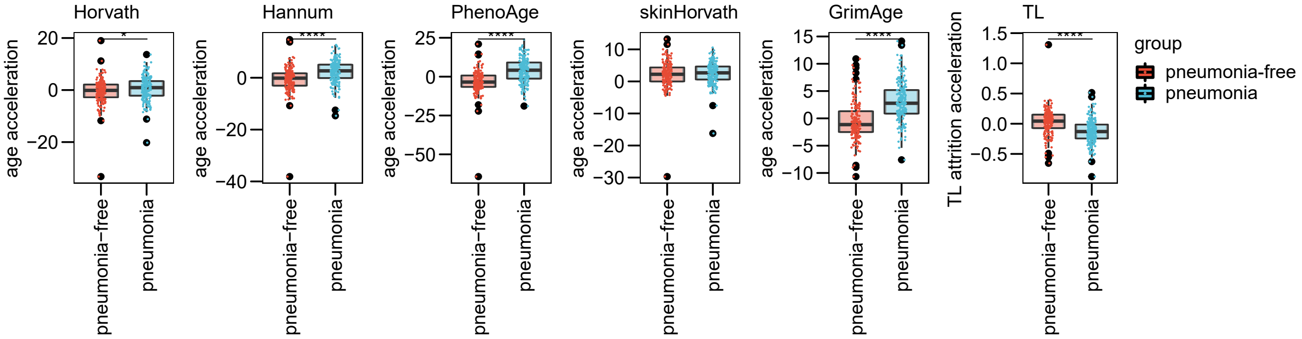

Supplementary figure 6 Epigenetic age acceleration in COVID-19 patients with or without pneumonia

The statistical significance for the t-test (*, p < 0.05; **, p <0.01; ***, p < 0.001; ****, p < 0.0001) is shown above the corresponding line.

Supplementary table 1 Demographic information of patient cohorts

	Healthy individuals	COVID-19 patients	p value
Age	41 (17-80)	43 (19-80)	0.179
Gender			0.229
Female	112 (48.3)	226 (53.1)	
Male	120 (51.7)	199 (46.9)	
