# Supplementary material for: Accelerated biological aging in COVID-19 patients
Source: Nat Commun. 2022 Apr 19;13:2135. doi: 10.1038/s41467-022-29801-8 (PMC9018863; doi:10.1038/s41467-022-29801-8)
Supplement: Supplementary file 2 — Reporting Summary [file 41467_2022_29801_MOESM2_ESM.pdf]

nature portfolio

Corresponding author(s): Huichuan Yu

Last updated by author(s): Feb 2, 2022

# Reporting Summary

Nature Portfolio wishes to improve the reproducibility of the work that we publish. This form provides structure for consistency and transparency in reporting. For further information on Nature Portfolio policies, see our Editorial Policies and the Editorial Policy Checklist.

## Statistics

For all statistical analyses, confirm that the following items are present in the figure legend, table legend, main text, or Methods section.

| n/a | Confirmed | |
|---|---|---|
| ☐ | ☒ | The exact sample size (*n*) for each experimental group/condition, given as a discrete number and unit of measurement |
| ☐ | ☒ | A statement on whether measurements were taken from distinct samples or whether the same sample was measured repeatedly |
| ☐ | ☒ | The statistical test(s) used AND whether they are one- or two-sided<br>*Only common tests should be described solely by name; describe more complex techniques in the Methods section.* |
| ☐ | ☒ | A description of all covariates tested |
| ☐ | ☒ | A description of any assumptions or corrections, such as tests of normality and adjustment for multiple comparisons |
| ☐ | ☒ | A full description of the statistical parameters including central tendency (e.g. means) or other basic estimates (e.g. regression coefficient) AND variation (e.g. standard deviation) or associated estimates of uncertainty (e.g. confidence intervals) |
| ☐ | ☒ | For null hypothesis testing, the test statistic (e.g. *F*, *t*, *r*) with confidence intervals, effect sizes, degrees of freedom and *P* value noted<br>*Give P values as exact values whenever suitable.* |
| ☒ | ☐ | For Bayesian analysis, information on the choice of priors and Markov chain Monte Carlo settings |
| ☒ | ☐ | For hierarchical and complex designs, identification of the appropriate level for tests and full reporting of outcomes |
| ☐ | ☒ | Estimates of effect sizes (e.g. Cohen's *d*, Pearson's *r*), indicating how they were calculated |

*Our web collection on statistics for biologists contains articles on many of the points above.*

## Software and code

Policy information about availability of computer code

| | |
|---|---|
| Data collection | The Illumina Infinium MethylationEPIC Beadchip (EPIC array) was used to profile genome-wide methylation using human blood sample. |
| Data analysis | All statistical analysis was performed using R Statistical Software (version 3.6.1) |

For manuscripts utilizing custom algorithms or software that are central to the research but not yet described in published literature, software must be made available to editors and reviewers. We strongly encourage code deposition in a community repository (e.g. GitHub). See the Nature Portfolio guidelines for submitting code & software for further information.

## Data

Policy information about availability of data

All manuscripts must include a data availability statement. This statement should provide the following information, where applicable:

- Accession codes, unique identifiers, or web links for publicly available datasets
- A description of any restrictions on data availability
- For clinical datasets or third party data, please ensure that the statement adheres to our policy

The complete DNA methylation raw data of the severe and non-severe COVID-19 cases have been deposited on the GEO repository under accession number GSE168739, and the clinical outcomes of these cases are not publicly available for data privacy but are available from Dr. Manel Esteller (mesteller@carrerasresearch.org) on request for research collaboration. The timeframe for response to data access requests is 30 days. There are no restrictions on the reuse of data. In addition, the DNA methylation raw data of the longitudinal cohort and healthy individuals analyzed in this study were available at GEO with identifiers of GSE161678, GSE149318, GSE123914, GSE145254, GSE118144 and GSE141682. Source data are provided with this paper.

# Field-specific reporting

Please select the one below that is the best fit for your research. If you are not sure, read the appropriate sections before making your selection.

- [x] Life sciences
- [ ] Behavioural & social sciences
- [ ] Ecological, evolutionary & environmental sciences

For a reference copy of the document with all sections, see nature.com/documents/nr-reporting-summary-flat.pdf

# Life sciences study design

All studies must disclose on these points even when the disclosure is negative.

| | |
|---|---|
| Sample size | This study included whole blood samples of 407 COVID-19 patients collected from fourteen hospitals in Spain (EBioMedicine 66, 103339 (2021)), six COVID-19 patients from Bernardes et al's longitudinal cohort in Germany (Immunity 53, 1296-1314.e9 (2020)) and 232 healthy individuals from previous studies with well-characterized populations (PLoS Genet. 16, e1009035 (2020); Epigenetics 13, 1056-1071 (2018); Transl Psychiatry 9, 118 (2019); Front Genet 10, 1188 (2019); Epigenetics 14, 341-351 (2019)). The sample size provided robust statistical power in the group comparison using correlation test and t-test. |
| Data exclusions | No data were excluded. |
| Replication | This is a comparative study in patient cohorts. The large sample size with replicating analysis using multiple epigenetic clocks may provide robust evidence as replicating measurement does. |
| Randomization | The subjects were grouped into three groups according to their illness and infection status: healthy individuals, non-severe COVID-19 and severe COVID-19 patients. Age and sex were matched between the COVID-19 and healthy groups. |
| Blinding | The investigators were blinded to group allocation in the primary analysis, while they were not blinded in the replicating analysis and results interpretation. |

# Reporting for specific materials, systems and methods

We require information from authors about some types of materials, experimental systems and methods used in many studies. Here, indicate whether each material, system or method listed is relevant to your study. If you are not sure if a list item applies to your research, read the appropriate section before selecting a response.

## Materials & experimental systems

| n/a | Involved in the study | |
|---|---|---|
| x | | Antibodies |
| x | | Eukaryotic cell lines |
| x | | Palaeontology and archaeology |
| x | | Animals and other organisms |
| | x | Human research participants |
| | x | Clinical data |
| x | | Dual use research of concern |

## Methods

| n/a | Involved in the study | |
|---|---|---|
| x | | ChIP-seq |
| x | | Flow cytometry |
| x | | MRI-based neuroimaging |

## Human research participants

Policy information about studies involving human research participants

| | |
|---|---|
| Population characteristics | The included COVID-19 patients did not present the risk factors of comorbidities, and the healthy individuals and COVID-19 patients did not present significant differences in age and gender. |
| Recruitment | Patients were eligible if they did not present the risk factors of comorbidities (obesity with a BMI 30, diabetes, hypertension, autoimmune disorders, and chronic cardiovascular or lung diseases). The whole blood samples of healthy individuals were collected before 2019 to ensure they had never been exposed to SARS-CoV-2. |
| Ethics oversight | The protocol of this study was approved by the institutional ethics review board of Josep Carreras Leukaemia Research Institute. Written informed consent was obtained from all participants. We conducted this study in compliance with the principles of the Declaration of Helsinki. |

Note that full information on the approval of the study protocol must also be provided in the manuscript.

# Clinical data

Policy information about clinical studies

All manuscripts should comply with the ICMJEguidelines for publication of clinical research and a completedCONSORT checklist must be included with all submissions.

| | |
|---|---|
| Clinical trial registration | This is not a clinical trial |
| Study protocol | This is not a clinical trial |
| Data collection | Whole blood samples and clinical data from patients with confirmed COVID-19 were collected between March 7th 2020 and September 14th 2020 from fourteen Hospitals in Spain. |
| Outcomes | we estimated the epigenetic age of the whole blood in COVID-19 patients and healthy individuals using the previously established epigenetic clocks (Hannum, Horvath, PhenoAge, skinHorvath and GrimAge clocks) and telomere length estimator. |
